# Antiviral factors and their counteraction by HIV-1: many uncovered and more to be discovered

**DOI:** 10.1093/jmcb/mjae005

**Published:** 2024-02-05

**Authors:** Dorota Kmiec, Frank Kirchhoff

**Affiliations:** In stitute of Molecular Virology, Ulm University Medical Center, 89081 Ulm, Germany; In stitute of Molecular Virology, Ulm University Medical Center, 89081 Ulm, Germany

**Keywords:** HIV-1, restriction factors, viral antagonists, interferon-stimulated genes, antiviral factors

## Abstract

Extensive studies on HIV-1 have led to the discovery of a variety of structurally and functionally diverse innate defense factors that target various steps of the retroviral replication cycle. Some of them, such as APOBEC3, tetherin, and SERINC5, are well established. Their importance is evident from the fact that HIV-1 uses its accessory proteins Vif, Vpu, and Nef to counteract them. However, the list of antiviral factors is constantly increasing, and accumulating evidence suggests that innate defense mechanisms, which restrict HIV-1 and/or are counteracted by viral proteins, remain to be discovered. These antiviral factors are relevant to diseases other than HIV/AIDS, since they are commonly active against various viral pathogens. In this review, we provide an overview of recently reported antiretroviral factors and viral countermeasures, present the evidence suggesting that more innate defense mechanisms remain to be discovered, and discuss why this is a challenging but rewarding task.

## Introduction

When HIV-1 was discovered ∼40 years ago (Barré-Sinoussi et al., 1983; Levy et al., 1984), it was commonly assumed that the ability of viruses to spread within specific cell types was largely determined by the expression of cell surface receptors required for viral entry. Thanks to the extensive research on HIV-1 and related primate lentiviruses, it has become clear that the intricate interplay between viruses and their host cells is much more complex than initially anticipated. While viruses exploit numerous cellular factors to propagate and spread, cells are not defenseless and have evolved numerous innate immune defense factors that can inhibit viral pathogens at every step of their replication cycle.

Almost 20 years after the discovery of HIV-1 as the causative agent of acquired immune deficiency syndrome (AIDS), researchers identified the first key restriction factor against this virus. In 2002, the Malim group reported that apolipoprotein B mRNA-editing enzyme catalytic polypeptide-like 3G (APOBEC3G) causes extensive G-to-A mutations in viral DNA during reverse transcription, rendering HIV-1 noninfectious (Sheehy et al., 2002). Two years later, the Sodroski group discovered that tripartite motif-containing protein 5 alpha (TRIM5α) targets retroviral capsids and interferes with the early stages of HIV-1 infection in a species-dependent manner (Stremlau et al., 2004). In 2008, the Bieniasz group and the Guatelli group independently reported that tetherin, also known as bone marrow stromal antigen 2, inhibits the release of newly formed HIV-1 particles by physically tethering them to the cell surface (Neil et al., 2008; Van Damme et al., 2008). Three years later, the Benkirane and Skowronski laboratories showed that SAM domain and HD domain-containing protein 1 suppresses HIV-1 replication in some cell types by reducing the pool of available deoxynucleoside triphosphates required for reverse transcription (Hrecka et al., 2011; Laguette et al., 2011). In 2015, the Göttlinger group and the Pizzato group independently showed that the incorporation of serine incorporator 5 (SERINC5) into HIV-1 virions impairs their ability to fuse with their target cells (Rosa et al., 2015; Usami et al., 2015).

These studies illustrate enormous structural, functional, and evolutionary diversity of restriction factors, which makes it impossible to establish universal defining criteria. While exceptions exist, common characteristics include inducibility by interferons (IFNs) or virus infection and interaction with viral components. In addition, restriction factors are among the most variable cellular proteins. Their amino acid changes are driven by positive selection pressure, prompting adaptations to counter emerging pathogens or evade viral countermeasures. This dynamic reflects the continuous evolutionary arms racing between viruses and their hosts (Kirchhoff, 2010; Duggal and Emerman, 2012; Harris et al., 2012; Malim and Bieniasz, 2012). Many antiviral genes, including APOBEC3 and TRIM5α, have not only diversified but also multiplied during mammalian evolution and now form families with distinct functions in innate immunity and beyond (Refsland and Harris, 2013; Salter et al., 2016; Müller and Sauter, 2023). Thus, antiviral factors synergize and may act in a redundant manner to reduce the risk of viral resistance. They are often expressed in specific cell types and usually exhibit broad activity against various viral pathogens and families (Kluge et al., 2015; Colomer-Lluch et al., 2018).

Viruses have evolved complex countermeasures to avoid effective innate control or elimination. The HIV-1 genome encodes four accessory proteins: viral infectivity factor (Vif), viral protein R (Vpr), viral protein U (Vpu), and negative factor (Nef). These proteins are often not essential for viral replication in cell lines but are critical for viral immune evasion and effective spread *in vivo*. Efficient counteraction by HIV-1 accessory proteins is perhaps the best-accepted evidence for the importance of an antiviral protein. For example, APOBEC3 proteins are targeted by Vif for degradation, preventing virion incorporation, and hence maintaining its genetic integrity (Sheehy et al., 2002). Several key enzymes in DNA repair pathways (UNG2, HTLF, and Exo1) are targeted for proteasomal degradation by Vpr, which is associated with viral particles (Hrecka et al., 2016; Lahouassa et al., 2016; Yan et al., 2018). Tetherin is counteracted by HIV-1 Vpu and simian immunodeficiency virus (SIV) Nef through distinct mechanisms that ultimately reduce its expression at the cell surface (Neil et al., 2008; Jia et al., 2009; Sauter et al., 2009; Zhang et al., 2009). Nef also downregulates SERINC5 expression on the cell surface to prevent virion incorporation (Rosa et al., 2015; Usami et al., 2015). SERINC5 illustrates that exceptions exist to all of the outlined characteristics, since it does not show a response to positive selection pressure and its expression is not induced by IFNs.

These well investigated and documented antiviral factors also play roles in viral sensing and cancer evolution (Hotter et al., 2013; Mauney and Hollis, 2018; Venkatesan et al., 2018). Recent studies have uncovered a substantial number of additional antiviral factors proposed to inhibit HIV-1 through distinct mechanisms. However, there are yet-unknown cellular factors that are induced by IFNs and involved in the sexual transmission of HIV-1, viral rebound after treatment interruption, and/or the resistance of some cell types to infection. This review provides a brief overview of the discovery and features of recently documented antiretroviral factors. In addition, we provide evidence showing that more factors remain to be discovered and discuss why this represents a significant challenge but seems highly warranted. In many cases, the physiological relevance and restrictive mechanisms of recently reported antiviral proteins, as well as potential viral countermeasures, need further investigation. Unless definitive evidence has been reported for antiviral mechanisms and viral countermeasures, we refer to cellular proteins reported to inhibit HIV-1 as ‘antiviral’ rather than ‘restriction’ factors.

## Novel antiretroviral factors discovered in the past decade

**Figure 1:**
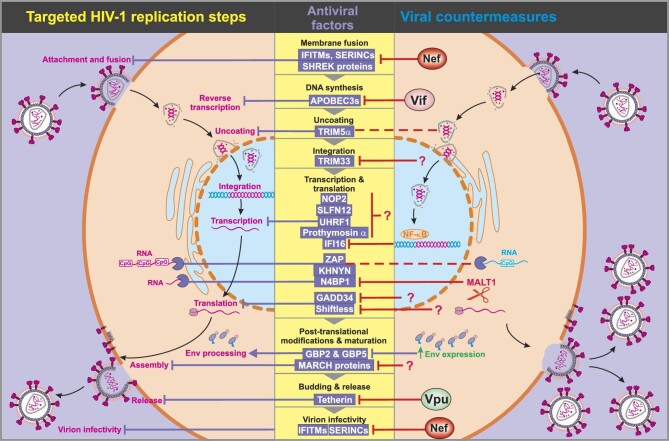
Overview of selected antiretroviral factors, viral replication steps that might be targeted, and viral countermeasures. A retroviral replication cycle is shown in the left panel. Please note that the list of antiviral factors is far from complete and the exact mechanisms and targets of most factors shown here remain to be fully defined.

The identification of novel antiviral factors has been the focus of numerous studies (Table 1), and the newly identified factors target essentially all steps of the retroviral replication cycle (Figure 1). While traditional screening approaches rely on the overexpression of genes of interest in transfected cell lines (Schoggins et al., 2011; McLaren et al., 2015; Kane et al., 2016), advancements in genome editing make it possible to assess antiviral effectors in cell types that are naturally susceptible to HIV-1 infection. A recent study with CRISPR/Cas9-mediated knockout of 426 genes in primary human CD4^+^ T cells revealed 23 factors with inhibitory effects on HIV-1 (Hiatt et al., 2022), including both known antiviral factors (EIF2AK2/PKR and G3BP1) and novel candidates (ATP2A2, ITGA4, NDUFB10, P4HB, LDLR, RBM17, RARS, SDCCAG8, SMG6, LARP7, MEPCE, AMBRA1, CUL2, HUWE1, HDAC3, NCOR1, COPS2, DDB1, VPRBP, CEP57, and RANBP2). Similarly, a study using a custom library targeting ∼500 IFN-stimulated genes (ISGs) revealed that HM13, IFI16, UBE2L6, IGFBP2, and LAP3 contribute to IFN-mediated restriction of HIV-1 in CD4^+^ T cells (Itell et al., 2023). Another elegant study employing an HIV-1 virion-packageable CRISPR screen performed in monocyte-derived THP-1 cells identified that IFN effectors, such as zinc finger antiviral protein (ZAP), TRIM25, NEDD4-binding protein 1 (N4BP1), MxB, IFITM1, UBE2L6, IFI16, tetherin, and TRIM5α, as well as non-ISGs could affect HIV-1 replication (OhAinle et al., 2018). The identification of known restriction factors through these screens is a sign of the effectiveness of CRISPR-based approaches. For example, IFI16, identified as a common hit in these studies, was initially found to inhibit HIV-1 in an overexpression screen for genes sharing characteristics of known restriction factors (McLaren et al., 2015). Follow-up studies revealed that IFI16 and related Pyrin and HIN domain-containing proteins, i.e. myeloid cell nuclear differentiation antigen (MNDA) and interferon-inducible protein X, restrict HIV-1 transcription by sequestering the host transcription factor Sp1 (Hotter et al., 2019; Bosso et al., 2020). Meanwhile, siRNA-mediated knockdown is also useful for identifying antiviral effectors. An RNAi screen of 17746 human genes performed in HeLa cells identified numerous factors affecting HIV-1 replication (Zhu et al., 2014). In addition to CCNK and BRD4, two known antiretroviral factors, the nucleolar m^5^C RNA methyltransferase NOP2 (also known as NSUN1) ranked as a top hit. NOP2 suppresses HIV-1 transcription and promotes viral latency (Kong et al., 2020). A similar screen in HeLa cells using a library of siRNAs against all components of the ubiquitin-conjugation machinery revealed that TRIM33 targets HIV-1 integrase for proteasomal degradation (Ali et al., 2019).

**Table 1:** Novel antiviral factors and proposed inhibitory mechanisms.

**Antiviral factors**	**Mechanism of action**	**Tested cell types**	**References**
C19orf66	Inhibits ribosomal frameshifting of HIV-1, leading to premature translation termination	HEK293T cells, HOS-CD4^+^-CCR5^+^ cells, MT4 cells, THP-1 cells, primary human monocyte-derived macrophages	Wang et al. (2019)
GADD34	Inhibits HIV-1 protein expression by viral 5′-UTR TAR RNA-mediated translational inhibition	HEK293T cells, CD4^+^ HeLa cells, MT-2 cells, Jurkat T cells	Ishaq et al. (2020)
GBP2, GBP5	Inhibit the cellular protease furin, which processes envelope glycoproteins of various viral pathogens	HEK293T cells, primary human lymphoid aggregate cultures, primary human monocyte-derived macrophages	Krapp et al. (2016); Braun et al. (2019)
KHNYN	Cooperates with ZAP to degrade CpG-enriched HIV-1 RNA	HeLa cells	Ficarelli et al. (2019)
MARCH1, MARCH2, MARCH8	Reduce Env incorporation into budding virions	HEK293T cells, H9 T cells, primary human monocyte-derived macrophages	Tada et al. (2015); Zhang et al. (2018)
N4BP1	Degrades viral mRNA in unstimulated T cells; is cleaved and inactivated by paracaspase MALT1 following T cell activation	HEK293T cells, Jurkat T cells, primary human monocyte-derived macrophages	OhAinle et al. (2018); Yamasoba et al. (2019)
NOP2	Suppresses HIV-1 transcription and promotes viral latency; competes with Tat for TAR RNA binding and leads to its m^5^C methylation	HEK293T cells, J-Lat A2 cells, MAGI-HeLa cells, Jurkat T cells, primary CD4^+^ T cells	Zhu et al. (2014); Kong et al. (2020)
Prothymosin α	Inhibits viral transcription	HEK293T cells, peripheral blood mononuclear cells from participants with acute HIV-1 infection	Geretz et al. (2023)
IFI16, PYHIN1, MNDA	Suppress HIV-1 gene expression through binding to transcription factor Sp1	HEK293T cells, THP-1 cells, monocyte-derived macrophages, primary CD4^+^ T cells	McLaren et al. (2015); Hotter et al. (2019); Bosso et al. (2020)
SLFN11, SLFN12	Cause a codon-usage-specific translational block to inhibit HIV-1 replication and reactivation	HEK293T cells, ACH2 cells	Li et al. (2012); Kobayashi-Ishihara et al. (2023)
SHREK protein family: PSGL-1, CD43, TIM-1, CD34, PODXL1, PODXL2, CD164, MUC1, MUC4, and TMEM123	Incorporate into virions and interfere with HIV-1 attachment to target cells	HEK293T cells, HeLa cells, Jurkat T cells, primary CD4^+^ T cells	Liu et al. (2019); Fu et al. (2020); Dabbagh et al. (2021); Burnie et al. (2022)
TRIM33	Degrades HIV-1 integrase through K48-linked polyubiquitination	SupT1 cells, HeLa cells	Ali et al. (2019)
UHRF1	Inhibits HIV-1 LTR-driven transcription through ubiquitination–proteasomal degradation of Tat	U1 cells, TZM-bl cells, Jurkat T cells, J-Lat 10.6 cells, A2 cells, primary CD4^+^ T cells	Liang et al. (2021)

Antiviral factors often directly interact with virus components. Thus, another group of candidates are cellular factors that interact with specific viral transcripts or proteins. Analyses of infected Jurkat T cells for HIV-1 RNA interactors revealed  >200 human proteins, including factors previously reported to impact HIV-1 replication. Although knockdown of LRPPRC, DHX30, MBOAT7, DLD, NCLN, and RBBP4 increased HIV-1 reporter gene expression, the functional mechanism and relevance of most interactions remain unclear (Knoener et al., 2017, 2021). A more focused screen of 62 cellular RNA-binding proteins identified N4BP1 as an inhibitor of HIV-1that degrades viral RNA in primary T cells and macrophages (Yamasoba et al., 2019).

A variety of factors are thought to interfere with HIV-1 gene expression. For example, ubiquitin-like with PHD and RING finger domain 1 (UHRF1) inhibits HIV-1 long terminal repeat (LTR)-driven transcription and promotes viral latency (Liang et al., 2021). Recent advancements in single-cell omics approaches have allowed the identification of antiviral factors in primary viral target cells in culture and even *in vivo*. Specifically, a single-cell transcriptomic approach was used to identify host factors associated with HIV-1 control during acute infection. The correlation of host gene expression with viral RNA abundance within individual cells from HIV-1-infected individuals identified prothymosin α as an inhibitor of viral transcription (Geretz et al., 2023). Schlafen 11 (SLFN11) and SLFN12 target the next step in the replication cycle, imposing codon-usage-specific translational blocks and inhibiting HIV-1 replication and reactivation (Li et al., 2012; Kobayashi-Ishihara et al., 2023). Similarly, ZAP inhibits HIV-1 at the post-transcriptional level (Gao et al., 2002) by binding to CpG dinucleotides in viral RNAs (Takata et al., 2017) and recruiting KH and NYN domain-containing endonuclease (KHNYN) to degrade them (Ficarelli et al., 2019). In 2019, a paralogue of KHNYN, N4BP1, was shown to restrict HIV-1 and be cleaved by MALT1 in activated CD4^+^ T cells (Yamasoba et al., 2019). Intriguingly, the antiviral activity of N4BP1 might be functionally linked to ZAP (OhAinle et al., 2018). While accumulating evidence suggests that ZAP inhibits HIV-1 through an antiviral complex rather than independently, it is also proposed to directly cause translational repression of viral transcripts (Zhu et al., 2012). Recently, C19orf66 (also known as Shiftless) was identified as an inhibitor of HIV-1 ribosomal frameshifting required for Gag–Pol expression (Wang et al., 2019). Finally, growth arrest and DNA damage-inducible protein 34 (GADD34) was shown to inhibit HIV-1 translation (Ishaq et al., 2020).

A large number of cellular factors have been reported to impair viral infectivity. Initially, IFITMs and SERINC5 were shown to interfere with the infectiousness of HIV-1 particles (Compton et al., 2014; Rosa et al., 2015; Usami et al., 2015). More recent studies demonstrated that membrane-associated RING-CH proteins (MARCH1, MARCH2, and MARCH8) reduce Env incorporation into budding virions (Tada et al., 2015; Zhang et al., 2018; Umthong et al., 2021). PSGL-1 and other members of the surface-hinged, rigidly-extended killer (SHREK) family (CD43, TIM-1, CD34, PODXL1, PODXL2, CD164, MUC1, MUC4, and TMEM123) were reported to suppress HIV-1 attachment to target cells (Liu et al., 2019; Fu et al., 2020; Dabbagh et al., 2021; Burnie et al., 2022). In contrast, guanylate-binding proteins (GBP2 and GBP5) impair HIV-1 infectivity by inhibiting the cellular protease furin, which cleaves the HIV-1 gp160 Env precursor into mature gp120 and gp41 (Krapp et al., 2016; Braun et al., 2019). Some HIV-1 strains evade this restriction by mutations in the start codon of the accessory *vpu* gene, increasing Env expression at the cost of Vpu function (Krapp et al., 2016; Yamada et al., 2018).

## Novel functions and targets of HIV-1 accessory proteins

The striking multi-functionality of HIV-1 accessory proteins has been the topic of previous reviews (Abraham and Fackler, 2012; Guenzel et al., 2014; González, 2017; Sauter and  Kirchhoff, 2018; Volcic et al., 2023), but accumulating evidence shows that it goes even further than anticipated (Table 2).

**Table 2:** Novel functions and targets of HIV-1 accessory proteins.

**HIV-1 accessory protein**	**Targets**	**Mechanism**	**Tested cell types**	**References**
Vif	PP2A	Vif induces CUL5-dependent proteasomal degradation of the cellular phosphatase PP2A	HIV-1-infected CD4^+^ T cells	Greenwood et al. (2016); Marelli et al. (2020)
	FMR1, DPH7	Unknown	HIV-1-infected CD4^+^ T cells	Naamati et al. (2019)
Vpr	SIRT7	Vpr promotes the degradation of SIRT7 by activating the host CRL4–DCAF1 E3 ligase	HEK293T cells	Zhou et al. (2021)
	Autophagy	Vpr promotes the enlargement and dysfunction of autophagosomes and lysosomes	Primary neuronal cells	Santerre et al. (2021)
	SLF2	Vpr depletes SLF2 and increases chromatin accessibility of unintegrated viral DNA	CD4^+^ T cells	Dupont et al. (2021)
Vpu	PSGL-1	Vpu downregulates PSGL-1 expression on the cell surface	CD4^+^ T cells	Fu et al. (2020)
	Peroxisome biogenesis	Vpu induces microRNAs that target mRNAs encoding proteins required for peroxisome formation and function	HIV-1-infected CD4^+^ T cells and monocyte-derived macrophages	Xu et al. (2020)
	UBE2L6, PLP2	Vpu mediates proteasomal degradation of UBE2L6 and PLP2	T cells, THP1 cells, HeLa cells, monocyte-derived macrophages	Jain et al. (2018)
	Tim-3	Vpu sequesters Tim-3 within the trans-Golgi network and Rab5^+^ compartments to downregulate Tim-3 expression on the cell surface	HIV-1-infected CD4^+^ T cells	Prévost et al. (2020)
	CD47	Vpu downregulates CD47 expression on the cell surface	HIV-1-infected CD4^+^ T cells	Cong et al. (2021)
Nef	PSGL-1	In addition to Vpu, Nef also downregulates PSGL-1 expression on the cell surface	HEK293T cells	Fu et al. (2020)
	SERINC3, SERINC5	Nef downregulates the expression of SERINC3 and SERINC5 on the cell surface	Monocyte-derived macrophages, T cells	Rosa et al. (2015); Usami et al. (2015)
	IFITMs	Unknown	Primary CD4^+^ T cells	Lee et al. (2018)
	Autophagy	Nef inhibits autophagy initiation as well as autophagosome and lysosome fusion	HIV-1-infected monocyte-derived macrophages and CD4^+^ T cells	Castro-Gonzalez et al. (2021)

Vif is the evolutionarily oldest lentiviral accessory protein and is already found in the genomes of prosimian and feline immunodeficiency viruses (Gifford, 2012). Vif is thought to have evolved to counteract the antiviral activity of APOBEC3 proteins (Nakano et al., 2017). To degrade APOBEC3 proteins, Vif hijacks the cullin–RING ubiquitin ligase (CRL) complex. Recently, proteomic analysis of T cells infected with wild-type or *vif*-deficient HIV-1 revealed that Vif degrades components of host PP2A phosphatase regulators (Greenwood et al., 2016; Marelli et al., 2020). Although the functional relevance is not fully understood, degradation of PP2A by Vif is a conserved process and leads to G2/M arrest thought to promote HIV-1 replication (Izumi et al., 2010). Independent proteomic analysis of infected primary CD4^+^ T cells identified FMR1 and DPH7 as additional targets of Vif (Naamati et al., 2019), among which, FMR1 has been shown to reduce HIV-1 infectivity (Pan et al., 2009).

Vpr is encoded by all primate lentiviruses and perhaps the most enigmatic HIV-1 accessory protein. Numerous effects of Vpr, including the enhancement of reverse transcriptase activity, nuclear import of the pre-integration complex, HIV-1 transcription, gene splicing, apoptosis, and cell cycle arrest, have been reported (Wallet et al., 2020). Vpr is found in virions and counteracts several cellular nucleases prior to proviral integration (Fabryova and Strebel, 2019). Recently proposed targets of Vpr include the repressor of HIV-1 transcription ZBTB2 (Bruce et al., 2021), the inhibitor of HIV-1 infectivity in macrophages LAPTM5 (Zhao et al., 2021), the antiviral PCIF1 methyltransferase (Zhang et al., 2021), and the RNA-associated early-stage antiviral factor RPRD2 (Liu et al., 2011; Gibbons et al., 2020). Vpr has also been reported to synergize with Nef to reduce the expression of the mannose receptor in macrophages (Lubow et al., 2020) and to interfere with autophagy (Santerre et al., 2021).

Vpu is only found in HIV-1 and its closest SIV relatives, infecting chimpanzees, gorillas, and some closely related *Cercopithecus* species. CD4 degradation and tetherin antagonism are the best-established functions of Vpu, and numerous additional activities have been reported (Volcic et al., 2023). For example, Vpu induces the expression of four microRNAs that target mRNAs encoding proteins required for peroxisome formation and metabolic function (Xu et al., 2020). Arrayed analysis of protein degradation revealed that Vpu targets CD99 and PLP2, which interfere with the packaging of HIV-1 Env and degrade the cellular E2 ligase UBE2L6 to inhibit the global conjugation of ISG15 (Jain et al., 2018). Vpu inhibits NF-κB activity, thereby suppressing the expression of thousands of cellular proteins, including interferons and antiviral factors (Langer et al., 2019). In addition, Vpu downregulates the antiviral protein Tim-3 on the surface of infected CD4^+^ T cells (Prévost et al., 2020). It has also been suggested that Vpu targets CD47 to enhance the capture and phagocytosis of CD4^+^ T cells by macrophages and to allow infection by CCR5-tropic T/F HIV-1 strains (Cong et al., 2021).

Nef is well known for its multi-tasking ability. Nef has attracted much research interest because the lack of Nef is associated with lower viral loads and slower disease progression (Kestler et al., 1991; Deacon et al., 1995; Kirchhoff et al., 1995). As mentioned above, Nef counteracts SERINC5 (Rosa et al., 2015; Usami et al., 2015). In activated CD4^+^ T cells, the lack of Nef-mediated SERINC antagonism only moderately impaired HIV-1 replication (Kmiec et al., 2018). However, in monocyte-derived macrophages, virion incorporation of SERINC5 potentiated the production of proinflammatory cytokines and strongly impaired virion infectivity (Pierini et al., 2021). Some HIV-1 strains with changes in the V3 loop of the external envelope glycoprotein gp120 or truncations in the transmembrane glycoprotein gp41 are largely resistant to SERINC5 even in the absence of Nef (Heigele et al., 2016; Beitari et al., 2017; Haider et al., 2021). It has been reported that Nef antagonizes IFITM proteins (Lee et al., 2018), but the effects are modest and need further investigation. Nef also impacts cytoplasmic proteins involved in cellular homeostasis and stress responses. For example, Nef was shown to counteract the antiviral activity of cellular stress-associated HDAC6 by inducing its degradation (Marrero-Hernández et al., 2019) and suppressing autophagy, which may otherwise target virions and/or viral components for degradation (Castro-Gonzalez et al., 2021).

## Additional antiviral factors remain to be discovered

While numerous antiviral factors have been reported, it is evident that additional factors, including ISGs and targets of HIV-1 accessory proteins, exist. For example, transmitted/founder (TF) HIV-1 strains responsible for primary infection are more resistant to IFNs than the viruses found during later stages of infection (Keele et al., 2008; Parrish et al., 2013). In some cases, Vpu-mediated counteraction of tetherin and/or resistance to IFITM-mediated restriction contributes to the resistance of TF HIV-1 infectious molecular clones (IMCs) to IFNs (Foster et al., 2016; Kmiec et al., 2016). Most mutations associated with IFN resistance in TF HIV-1 strains, however, do not affect viral sensitivity to well-established antiretroviral restriction factors. Recently, it was shown that the HIV-1 strains responsible for viral rebound after treatment interruption share IFN resistance with TF HIV-1 strains (Gondim et al., 2021), but the ISGs that prevent viral rebound are largely unknown.

Functional analyses showed that TF HIV-1 M particles are more efficiently released from infected cells than from chronic IMCs, and this difference was only partly dependent on Vpu (Kmiec et al., 2016). Thus, HIV-1 has evolved additional yet-to-be-defined Vpu-independent functions to promote efficient virus release and replication in the face of an innate antiviral response. In addition, Nef enhances HIV-1 replication in cells lacking SERINC3 and SERINC5, suggesting other antiviral factor that can be counteracted by Nef (Olety et al., 2023). Indeed, network mapping studies suggest that each of HIV-1 accessory proteins interacts with a large number of cellular proteins (Jäger et al., 2012; Hiatt et al., 2022). Finally, HIV-1 infection induces numerous post-translational modifications, such as phosphorylation and ubiquitination, of cellular proteins, and a significant portion of these modifications seem to be dependent on viral accessory proteins (Johnson et al., 2022). Thus, several lines of evidence suggest that relevant innate antiviral mechanisms and viral countermeasures remain to be discovered.

## Challenges in defining and discovering novel restriction factors

The high structural and functional diversity of antiviral factors makes it highly challenging to discover novel factors. IFNs induce the expression of hundreds, if not thousands, of cellular factors that are often poorly characterized. Antiviral factors that are not ISGs are even more difficult to discover. For example, Nef is known to increase virion infectivity (Chowers et al., 1994; Miller et al., 1994), and it took >20 years to identify SERINC5 as an antiviral factor that impairs HIV-1 infectivity and is counteracted by Nef (Rosa et al., 2015; Usami et al., 2015). It is also a highly challenging (if not impossible) task to unambiguously define antiviral restriction factors. The most straightforward definition would be cellular factors that significantly limit viral replication in relevant target cells. However, the most successful and thus best-studied viruses, such as pandemic HIV-1 strains, have been well adapted to their host and highly effective in counteracting or evading antiviral immune defenses. Therefore, restriction factors, especially those expressed in major viral target cells, may have little or no effect on the replication of wild-type HIV-1 strains with functional accessory genes. While IFNs inhibit HIV-1 in cell culture, therapeutic administration of IFNα to HIV-1-infected patients only induces a transient reduction in plasma viral loads. In fact, studies of chronic HIV-1 infection in humans and SIV infection in macaque models revealed positive correlations between viral loads and elevated plasma levels of IFNs and ISGs (Doyle et al., 2015). Thus, the inhibitory effect of major antiviral factors is mainly evident for pandemic HIV-1 M strains lacking accessory genes, their less-adapted group N, O, or P relatives or simian counterparts, and cell types that are not the main targets of the virus *in vivo*. In fact, the evolution of effective viral antagonists or evasion mechanisms is considered a better criterion than significant inhibitory effects in primary viral target cells for the importance of an antiviral factor. Paradoxically, the term ‘restriction’ factor is therefore most commonly used for cellular factors that do not potently restrict HIV-1 under physiological conditions because they are efficiently counteracted by the virus. Additionally, a primary antiviral function is difficult to define, because many antiviral factors also play physiological roles and their effects might be indirect. Furthermore, viruses hijack numerous cellular factors and pathways for replication. Thus, depletion or overexpression of cellular proteins involved in trafficking, transcription, metabolism, survival, or proliferation may exert inhibitory effects on viral replication. Consequently, some ‘antiviral’ hits may misleadingly appear, while others may be missed because HIV-1 has become very good at counteracting/evading them.

As mentioned above, screens using ISG expression libraries, RNA interference, or pooled CRISPR/Cas9 allowed the discovery of a variety of antiviral factors (Jones et al., 2021). However, genome-wide overexpression screens are prone to artefacts. RNAi screens identified relevant restriction factors. However, inefficient knockdown and off-target effects may affect reproducibility and lead to false positive (Jones et al., 2021). Most CRISPR/Cas9-based screens involve the introduction of pooled sgRNAs into Cas9-expressing cells, primarily via lentiviral transduction. Subsequently, cells showing resistance or sensitivity in single-round virus infection assays are enriched to identify proviral or antiviral factors, respectively, by next-generation sequencing. A targeted CRISPR-based screen for HIV restriction factors has been reported (OhAinle et al., 2018). However, this system is based on the co-packaging of vectors co-expressing Cas9 and sgRNAs. Thus, it only allows single rounds of replication for selection and only detects antiviral factors associated with altered levels of viral RNA genomes in the cell culture supernatant compared with the proviral copy numbers in the cells. Altogether, published genetic screens provide relevant insights into virus–host interactions, but their specificity, sensitivity, efficiency, and versatility are limited. Very recently, screens based on replication-competent HIV-1, Cytomegalo virus, and Influenza virus constructs expressing sgRNAs that modulate the activity of their cellular genes in Cas9-expressing cells have been reported (Bozzo et al., 2023; Finkel et al., 2023; King et al., 2023). Since the viral pathogens themselves drive the screens and the effects are boosted during each round of replication, these approaches are highly sensitive and may help substantially elucidate complex virus–host interactions and antiviral defense mechanisms.

## Relevance and perspectives

Restriction factors are the first line of defense protecting humans against most viral exposures, especially viral zoonoses. Similar to combined antiretroviral therapy, cellular antiviral factors, such as TRIM5α, APOBEC3 proteins, IFI16, ZAP, GBP2, GBP5, tetherin, and SERINC5, target different steps of the viral replication cycle, e.g. uncoating, reverse transcription, viral gene expression, viral RNA production, Env maturation, virion release, and infectivity (Figure 1). Intrinsic immunity factors have evolved to exert broad antiviral activity and frequently target conserved viral components. Thus, compared with therapeutics, they have the advantage of being active against many viral pathogens. Accumulating evidence suggests that other types of IFNs are more effective against viral pathogens than IFNα-2, which is commonly used for treatment (Lavender et al., 2016; Hayn et al., 2021). Thus, a better understanding of the induction and function of innate antiviral factors may allow us to reduce the side effects of IFN-based treatments while maintaining antiviral efficacy. In addition, understanding how individual antiviral factors suppress not only HIV-1 but also related endogenous retroviral elements and lentiviral vectors is required to optimize novel promising medical treatments, such as xenotransplantology, as well as gene- and stem cell-based therapies (Niu et al., 2017; Wu et al., 2019; Coroadinha, 2023).

Alternative ways to harness antiviral factors for therapeutic approaches against HIV-1 include the inhibition of viral antagonists or genetic editing to render antiviral factors resistant to viral antagonists. Several inhibitors of Vif, Vpu, and Nef have been reported (Emert-Sedlak et al., 2016; Robinson et al., 2022; Yan et al., 2022). Introduction of the gene encoding the rhesus macaque TRIMCyp into the germline protected cats against feline retroviruses (Wongsrikeao et al., 2011). The tandem APOBEC3C protein was designed to be a ‘super restriction factor’ that largely escapes antagonism by HIV-1 Vif (McDonnell et al., 2020). Although the therapeutic potential of innate antiviral factors holds great promise, they (except for IFN treatment) are still in the experimental stages due to significant challenges with regard to efficacy and safety. Considerable progress has been made in the development of high-throughput and comprehensive screening techniques and data analyses. Multi-omics approaches, such as genomics, transcriptomics, proteomics, and metabolomics, will allow us to obtain a more comprehensive view of host–virus interactions and antiviral defense mechanisms. In combination with single-cell analyses, these methods may allow the identification of cell-specific antiviral factors and determine which of them are most relevant for viral replication and persistence in infected individuals. In addition, functional genomics approaches, such as CRISPR/Cas9 knockout or activation screens, hold great promise for the identification of antiviral host factors. Progress in imaging technologies and structural biology techniques will allow us to better define the underlying mechanisms, and the increasing utilization of organoids or three-dimensional culture systems will help to ensure the relevance of the studied antiviral factors. The enormous capacity for sequencing in combination with rapid improvements in bioinformatics, computational biology, and artificial intelligence approaches should allow us to obtain in-depth insights into virus–host co-evolution and to better predict the risks of viral outbreaks and future pandemics.

The above-mentioned and other innovative approaches will expand our knowledge of innate immune defense mechanisms and almost certainly uncover new avenues for preventive, therapeutic, and curative strategies. Notably, these studies will have relevance beyond viral infections because restriction factors also play roles in maintaining cellular homeostasis, immune sensing and regulation, regulation of cellular proliferation, and tumor suppression, as well as autoimmune diseases and other immune disorders. Thus, antiviral factors play a role in central biological processes and have implications for various fields, including immunology and cancer research.

While enormous progress has been made in understanding antiviral factors, multiple questions remain. Many studies have reported the effect of IFNs on the induction of antiviral factors, but the precise molecular mechanisms and regulation of the antiviral activity remain to be determined. Furthermore, the functional redundancies and synergistic effects of antiviral factors, as well as their relative importance in different tissues and cell types, are poorly understood. Accumulating evidence shows that innate immune responses are tightly linked with adaptive immunity, but the underlying determinants are largely unclear. A better understanding of these interrelationships will be essential to unravel the co-evolutionary dynamics of virus–host interactions. To better assess the risk of future zoonoses, it is also important to examine the role of antiviral factors in non-human viral reservoirs and determine the species specificity. Previous studies suggested that the acquisition of a functional APOBEC3 antagonist is critical for the transmission of primate lentiviruses from monkeys to chimpanzees (Etienne et al., 2013, 2015) and effective tetherin antagonism contributes to the spread of pandemic HIV-1 M strains (Jia et al., 2009; Sauter et al., 2009). Other evidence supports the role of IFN-inducible antiviral factors in HIV-1 transmission and rebound after treatment interruption (Keele et al., 2008; Parrish et al., 2013; Gondim et al., 2021). Altogether, the impacts of specific antiviral factors on HIV-1 transmission, latency, and pathogenesis are still largely unknown. Addressing these issues will not only deepen our understanding of antiviral defense mechanisms but also hopefully allow us to harness antiviral factors for preventive, therapeutic, and curative purposes.

## Notes

**Author contributions:** D.K. and F.K. wrote the manuscript. D.K. drafted the figure, and F.K. modified and revised it.
